# Effect of the particle-hole channel on BCS–Bose-Einstein condensation crossover in atomic Fermi gases

**DOI:** 10.1038/srep25772

**Published:** 2016-05-17

**Authors:** Qijin Chen

**Affiliations:** 1 Department of Physics and Zhejiang Institute of Modern Physics, Zhejiang University, Hangzhou, Zhejiang 310027, China; 2 Synergetic Innovation Center of Quantum Information and Quantum Physics, Hefei, Anhui 230026, China

## Abstract

BCS–Bose-Einstein condensation (BEC) crossover is effected by increasing pairing strength between fermions from weak to strong in the particle-particle channel, and has attracted a lot of attention since the experimental realization of quantum degenerate atomic Fermi gases. Here we study the effect of the (often dropped) particle-hole channel on the zero *T* gap Δ(0), superfluid transition temperature *T*_c_, the pseudogap at *T*_c_, and the mean-field ratio 2Δ(0)/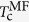, from BCS through BEC regimes, using a pairing fluctuation theory which includes self-consistently the contributions of finite-momentum pairs and features a pseudogap in single particle excitation spectrum. Summing over the infinite particle-hole ladder diagrams, we find a complex dynamical structure for the particle-hole susceptibility *χ*_*ph*_, and conclude that neglecting the self-energy feedback causes a serious over-estimate of *χ*_*ph*_. While our result in the BCS limit agrees with Gor’kov *et al*., the particle-hole channel effect becomes more complex and pronounced in the crossover regime, where *χ*_*ph*_ is reduced by both a smaller Fermi surface and a big (pseudo)gap. Deep in the BEC regime, the particle-hole channel contributions drop to zero. We predict a density dependence of the magnetic field at the Feshbach resonance, which can be used to quantify *χ*_*ph*_ and test different theories.

BCS–Bose-Einstein condensation (BEC) crossover has been an interesting research subject since 1980’s^1^,^2^,^3^,^4^,^5^,^6^,^7^,^8^,^9^,^10^,^11^,^12^,^13^,^14^,^15^,^16^. The experimental realization of BCS-BEC crossover in ultracold atomic Fermi gases^17^,^18^,^19^,^20^,^21^, with the help of Feshbach resonances, has given it a strong boost over the past decade^22^,^23^,^24^,^25^,^26^,^27^. When the pairing interaction is tuned from weak to strong in a two component Fermi gas, the superfluid behavior evolves continuously from the type of BCS to that of BEC^1^,^2^,^28^.

In such a fundamentally fermionic system, superfluidity mainly concerns pairing, namely, interactions in the particle-particle channel. In contrast, the particle-hole channel mainly causes a chemical potential shift, and is often neglected^29^. For example, in a conventional superconductor, the chemical potential below and above *T*_c_ are essentially the same, and thus its dependence on the temperature and the interaction strength has been completely neglected in the weak coupling BCS theory for normal metal superconductors. On the other hand, Gor’kov and Melik-Barkhudarov (GMB)^30^ considered the lowest order correction from the particle-hole channel, (which has been referred to as induced interaction in the literature), and found that both *T*_c_ and zero temperature gap Δ(0) are suppressed by a *big* factor of (4e)^1/3^ ≈ 2.22. Berk and Schrieffer^31^ also studied a similar effect in the form of ferromagnetic spin correlations in superconductors. Despite the big size of the GMB correction, the effect of the particle-hole channel has been largely overlooked in the theoretical study of BCS-BEC crossover, until it has become realistic to achieve such crossover experimentally in atomic Fermi gases. Heiselberg and coworkers^32^ considered the effect of the lowest order induced interaction in dilute Fermi gases and generalized it to the case of multispecies of fermions as well as the possibility of exchange of bosons. Kim *et al*.^33^ considered the lowest order induced interactions in optical lattices. Within the *mean-field* treatment and *without* including the excitation gap in the particle and hole propagators, these authors found the same effective overall interaction at zero *T* and at *T*_c_ and hence an unaffected mean-field ratio 2Δ(0)/*k*_B_*T*_c_ ≈ 3.53. Martikainen *et al*.^34^ considered the lowest order induced interactions in a three-component Fermi gas. It has become clear that including only the perturbative lowest order induced interaction is *not* appropriate away from the weak coupling BCS regime. Yin and coworkers^35^ went beyond the lowest order and considered the induced interactions from all particle-hole ladder diagrams, i.e., the entire particle-hole *T*-matrix. However, in all the above works, only the *bare* particle-hole susceptibility 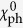 was considered, and it was averaged on-shell and only on the Fermi surface, with equal momenta for the particle and the hole propagators. No self-energy feedback was included. Therefore, there was necessarily no pseudogap in the fermion excitation spectrum at *T*_c_. This is basically equivalent to replacing the particle-hole susceptibility 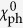 by an essentially temperature independent constant, leading to a simple downshift in the pairing interaction.

As the gap and *T*_c_ increase with interaction strength, it can naturally be expected that the contribution from the particle-hole channel, or the induced interaction, will acquire a significant temperature dependence. More importantly, *the presence of the (pseudo)gap serves to suppress the particle-hole fluctuations* (which tend to break pairing). In other words, neglecting the feedback of the gap related self energy in the particle-hole susceptibility is expected to cause an over-estimate of the particle-hole channel contributions. Therefore, a proper treatment should include the gap effect in the particle-hole susceptibility. In addition, the lowest order treatment is no longer appropriate away from the weak coupling regime.

Furthermore, it has now been established that as the pairing interaction increases, pseudogap develops naturally^12^,^28^,^36^. Experimental evidence for its existence comes from high *T*_c_ superconductors^13^,^28^,^37^,^38^,^39^ as well as atomic Fermi gases^40^,^41^,^42^,^43^,^44^. Therefore, a theory with proper treatment of the pseudogap effect is necessary in order to arrive at results that can be *quantitatively* compared with experiment. For the same reason, the effect of the particle-hole channel needs also to be studied within such a theory.

There have also been various quantum Monte Carlo (QMC) simulations^45^,^46^,^47^,^48^,^49^,^50^ on atomic Fermi gases, which includes both particle-particle and particle-hole channels, with an emphasis on the unitary limit. Some recent works^49^,^50^ seem to have produced good numbers when compared with experiment^51^. However, due to the black-box nature of QMC for non-specialists, these approaches do not provide physical understanding which is as transparent as an analytical theory, not to mention that there are large discrepancies between these QMC results^52^. Therefore, it is always important to develop a proper analytical theory.

In this paper, we explore the particle-hole channel effect based on a pairing fluctuation theory^10^,^53^, originally developed for treating the pseudogap phenomena of high *T*_c_ superconductors. This theory has been successfully applied to atomic Fermi gases and has been generating results that are in good agreement with experiment^12^,^28^,^40^,^42^. Here we include the entire particle-hole *T*-matrix, with gap effect included in the fermion Green’s functions. Instead of a simple average of the particle-hole susceptibility *χ*_ph_ on the Fermi surface, here we choose to average at two different levels – one on the Fermi surface, one over a narrow momentum shell around the Fermi level. We find that *χ*_ph_ has very strong frequency and momentum as well as temperature dependencies. It is sensitive to the gap size. Therefore, self-consistently including the self-energy feedback is important. For both levels of average, we find that while in the BCS limit, the particle-hole channel effect may be approximated by a downshift in the pairing strength so that the ratio 2Δ(0)/*T*_c_ is unaffected, the situation becomes more complex as the interaction becomes stronger where the gap is no longer very small. Significant difference exists for these two levels of averaging. The particle-hole susceptibility is reduced by both a smaller Fermi surface and a big (pseudo)gap in the crossover regime. Deep in the BEC regime, the particle-hole channel contributions drop to zero. Without including the incoherent part of the self energy, we find that at unitarity, *T*_c_/*E*_F_ ≈ 0.217, in reasonable agreement with experiment.

We emphasize that our theory is *not* a diagrammatic approach. Instead, it is derived using equations of motion^53^,^54^,^55^,^56^, and is simply recast in the form of Feynman diagrams for easy understanding. This also explains why we have self-energy feedback included in the diagrams.

The rest of this paper is arranged as follows. In the next section, we first give a brief summary of the pairing fluctuation theory without the particle-hole channel effect. Then we derive the theory with particle-hole channel included, starting by studying the dynamic structure of the particle-hole susceptibility. Next, we present numerical results, showing the effect of the particle-hole channel on the zero *T* gap, transition temperature *T*_c_ and pseudogap at *T*_c_, as well as the mean-field ratio 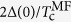. We also discuss and compare our value of *T*_c_/*E*_F_ with experiment and those in the literature. Finally, we conclude. More detailed results on the dynamic structure of the particle-hole susceptibility are presented in the Supplementary Info.

## Pairing Fluctuation Theory with the Particle-hole Channel Effect Included

### Summary of the pairing fluctuation theory without the particle-hole channel effect

To make this paper self-contained and to introduce the assumptions as well as the notations, we start by summarizing the pairing fluctuation theory^10^,^53^ without the effect of the particle-hole channel, as a foundation for adding the particle-hole channel.

**Figure 1:**
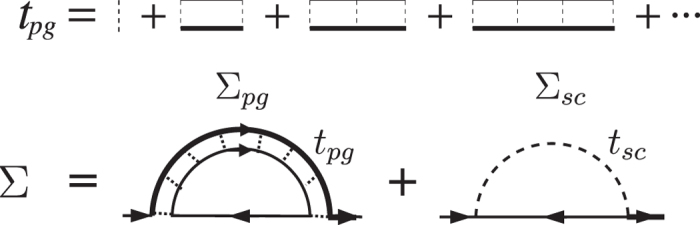
Feynman diagrams for the particle-particle channel *T*-matrix *t*_pg_ and the self energy Σ(*K*). The dotted lines represent the bare pairing interaction *U*. The dashed line, *t*_sc_, represents the superfluid condensate.

We consider a Fermi gas with a short range *s*-wave interaction *U*(**k**, **k**′) = *U* < 0, which exists only between opposite spins. Our theory can be effectively represented by a *T*-matrix approximation, shown diagrammatically in Fig. 1. However, *we emphasize that* Fig. 1 *is simply a representation of the equations derived from an equation of motion approach*^56^. This explains why we have fully dressed Green’s functions in the diagrams, *unlike a typical diagrammatic approach*. The self energy Σ(*K*) comes from two contributions, associated with the superfluid condensate and finite momentum pairs, respectively, given by Σ(*K*) = Σ_sc_(*K*) + Σ_pg_(*K*), where


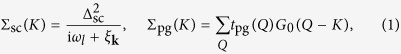


with Δ_sc_ being the superfluid order parameter. We use a four-vector notation, *K* ≡ (**k**, i*ω*_*l*_), *Q* ≡ (**q**, *i*Ω_*n*_), 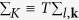, etc., and *ω*_*l*_ and Ω_*n*_ are odd and even Matsubara frequencies for fermions and bosons, respectively. Here *G*_0_(*K*) = (i*ω*_*l*_ − *ξ*_**k**_)^−1^ and 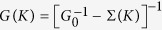 are the bare and full Green’s functions, respectively, *ξ*_**k**_ = *ħ*^2^*k*^2^/2*m* − *μ* is the free fermion dispersion, measured with respect to the Fermi level. In what follows, we will set *k*_B_ = *ħ* = 1. The pseudogap *T*-matrix


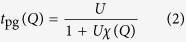


can be regarded as the renormalized pairing interaction with pair momentum *Q*, where


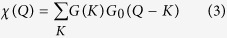


is the pair susceptibility. We emphasize that this asymmetric form of *χ*(*Q*) is not an *ad hoc* choice but rather a natural result of the equation of motion method. The bare Green’s function *G*_0_ comes from the inversion of the operator 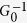 which appears on the left hand side of the equations of motion. It also appears in the particle-hole susceptibility *χ*_ph_, as will be shown below. Albeit not a phi-derivable theory, the equation of motion method ensures that this theory is more consistent with the Hamiltonian than a phi-derivable theory.

The gap equation is given by the pairing instability condition,


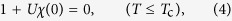


referred to as the Thouless criterion, which can also be naturally interpreted as the Bose condensation condition for the pairs, since 1 + *Uχ*(0) ∝ *μ*_pair_. In fact, after analytical continuation iΩ_*n*_ → Ω + i0^+^, one can Taylor expand the (inverse) *T*-matrix as


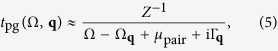


and thus extract the pair dispersion Ω_**q**_ ≈ *q*^2^/2M^*^ to the leading order, where M^*^ is the effective pair mass. Here Γ_**q**_ is the imaginary part of the pair dispersion and can be neglected when pairs become (meta)stable^10^,^53^,^56^. In the superfluid phase, *t*_pg_(*Q*) diverges at *Q* = 0 and a macroscopic occupation of the *Q* = 0 Cooper pairs, i.e., the condensate, appears. This macroscopic occupation, has to be expressed as a singular term, 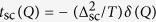, (the dashed line in Fig. 1), such that 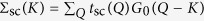, written in the same form as its pseudogap counterpart, Σ_pg_(*K*).

Now we split Σ_pg_(*K*) into coherent and incoherent parts:


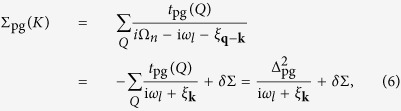


where we have defined the pseudogap Δ_pg_ via


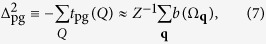


where *b*(*x*) is the Bose distribution function. Below *T*_c_, the divergence of *t*_pg_(*Q* = 0) makes it a good mathematical approximation to neglect the incoherent term *δ*Σ so that


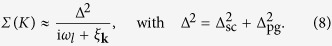


Therefore, the Green’s function *G*(*K*), the quasiparticle dispersion 
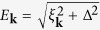
, and the gap equation, as expanded from Eq. (4), follow the same BCS form, *except that the total gap* Δ *now contains both contributions from the order parameter* Δ_sc_ *and the pseudogap* Δ_pg_.

For a contact potential, we get rid of the interaction *U* in favor of the scattering length *a* via 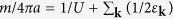, where *ε*_**k**_ = *k*^2^/2*m*. Then the gap equation can be written as


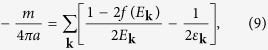


where *f*(*x*) is the Fermi distribution function. In addition, we have the particle number constraint, *n* = 2∑_*K*_*G*(*K*), i.e.,


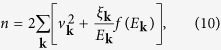


where 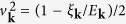 is the BCS coherence factor.

Equations (9), (10), and (7) form a closed set. For given interaction 1/*k*_F_*a*, they can be used to solve self consistently for *T*_c_ as well as Δ and *μ* at *T*_c_, or for Δ, Δ_sc_, Δ_pg_, and *μ* as a function of *T* below *T*_c_. Here *k*_F_ is the Fermi wave vector. More details about the Taylor expansion of the inverse *T* matrix can be found in refs 56 and 57.

### Dynamic structure of the particle-hole susceptibility *χ*_ph_(*P*)

**Figure 2:**
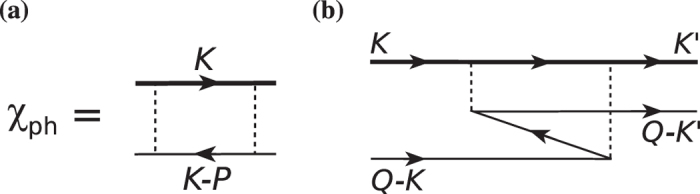
Feynman diagrams for the particle-hole susceptibility *χ*_ph_ in the presence of self-energy feedback effect. Panel (**b**) is identical to panel (**a**), with twisted external legs. The total particle-hole momentum *P* in (**a**) is equal to *K* + *K*′ − *Q* in (**b**), with *Q* being the particle-particle pair momentum.

Before we derive the theory with full particle-hole *T*-matrix *t*_ph_ included, we first study the dynamic structure of the particle-hole susceptibility *χ*_ph_(*P*). It is the single rung of the particle-hole scattering ladder diagrams, as shown in Fig. 2(a). Note that direct interaction exists only between fermions of opposite spins. Therefore, the particle and hole must also have opposite spins. The total particle-hole four-momentum is given by *P* ≡ (i*ν*_*n*_, **p**). Since we are considering the effect on pairing induced by the particle-hole channel, we can twist external legs of the diagram, as shown in Fig. 2(b), so that the particle-hole contribution can be added to the original pairing interaction *U* directly. It is obvious that the particle-hole momentum *P* in Fig. 2(a) is equal to *K* + *K*′ − *Q* in Fig. 2(b), where *Q* is the pair momentum of the particle-particle channel. Therefore, we have


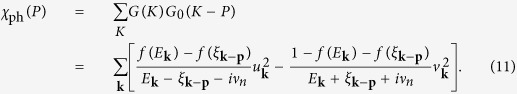


Note that again we have a mixing of dressed and undressed Green’s function in *χ*_ph_(*P*), like in the expression of *χ*(*Q*). As mentioned earlier, this mixing has exactly the same origin in both cases^56^. For convenience, here we dress the particle propagator with self energy and leave the hole propagator undressed. This is based on the fact that the hole propagator is undressed in *χ*(*Q*). (One can equivalently dress the hole while leaving the particle undressed).

A few remarks are in order. Firstly, the induced interactions conform to the Galileo transformation. Indeed, taking ±*K* and ±*K*′ as the four momenta of the incoming and outgoing fermions in the center-of-mass (COM) reference frame, the momenta in Fig. 2(b) should be relabeled as ±*K* + *Q*/2 and ±*K*′ + *Q*/2, so that *P* = (*K* + *Q*/2) − (−*K*′ + *Q*/2) = *K* + *K*′, independent of *Q*, same as in the COM frame. Secondly, as in the Noziéres and Schmitt-Rink (NSR) theory^2^, one needs a fictitious separable potential *U*_**k,k**′_ = *Uφ*_**k**_*φ*_**k**′_, such as the contact potential considered for atomic Fermi gases, in order to have a simple result in the form of Eq. (2) for the summation of the particle-particle ladder diagrams^8^,^10^,^53^. However, inclusion of the particle-hole channel spoils this separability for the total effective interaction *U*_eff_(**k, k**′), since *χ*_ph_(*P*) only depends on the sum *P* = *K* + *K*′.

Upon analytical continuation, i*ν*_*n*_ → *ν* + i0^+^, we separate the retarded 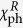 into real and imaginary parts, 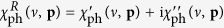, where


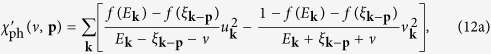



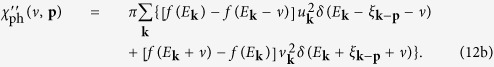


It is easy to see 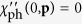, and 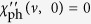 if 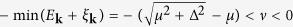 or 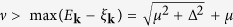. At low *T*, 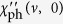 is gapped; it is exponentially small for |*ν*| < Δ if *μ* > 0 or for 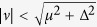 otherwise. In all cases, 
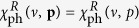
 is isotropic in **p**. In the BCS limit, Δ → 0, *E*_**k**_ → |*ξ*_**k**_|, so that


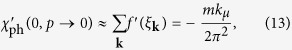


where 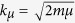 is the momentum on the Fermi surface.

For comparison, we analyze the undressed particle-hole pair susceptibility,


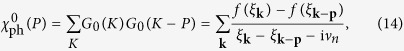


which is studied by GMB^30^ and others^32^,^33^,^34^,^35^ in the literature.

The imaginary part is given by


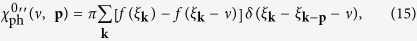


with 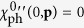. For *ν* ≠ 0, 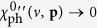 exponentially as *p* → 0. For *small* but finite *p*,


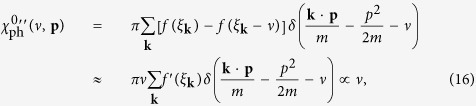


where the delta function can be satisfied only for 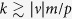. When |*ν*|*m*/*p* > *k*_*μ*_, we have *ξ*_**k**_ > 0 so that 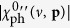 will also turn around and start to decrease exponentially. The turning points *ν* =  ± *pk*_*μ*_/*m* show up as two peaks in 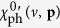, which satisfies


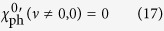


and


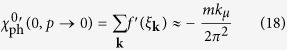


at low *T*. More generally, for *ν* = 0 and finite *p*, we have


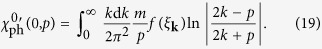


In the weak coupling limit, 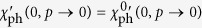, since *χ*_ph_ reduces to 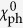 when the gap Δ vanishes.

**Figure 3:**
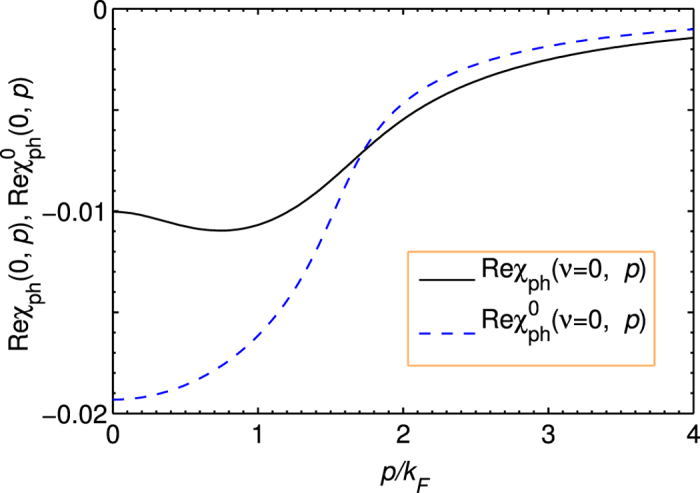
Strong momentum dependence of the real part of the particle-hole susceptibility at zero frequency *ν* = 0 in the unitarity limit, with (black curve) and without (blue dashed curve) self-energy feedback, calculated at *T* = 0.3*T*_c_, where *T*_c_ = 0.256*E*_F_. While the undressed 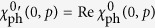 shows a simple monotonic behavior, the dressed susceptibility 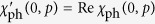 has a nonmonotonic *p* dependence, and a substantially reduced value at *p* = 0. This reduction derives from the gap effect in the Green’s function *G*(*K*). Namely, 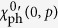 seriously over-estimated particle-hole fluctuations.

It is easy to show that the hermitian conjugate 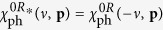. Similar relations do not hold for *χ*_ph_, however, due to the mixing of *G*_0_ and *G* in the expression of *χ*_ph_(*P*). Such symmetry relations are manifested in Supplementary Figs S1–S3, which show three- and two-dimensional plots of the real and imaginary parts of 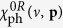 and 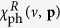 at different *T* at unitarity. These plots reveal that by neglecting the feedback effect, the bare 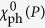 misses important interesting dynamic structures associated with the pseudogap, which leads to a low frequency gap in 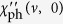. This gap becomes wider at lower *T*. The evolution of 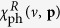 with temperature, 1/*k*_*F*_*a*, and momentum *p* is shown in Supplementary Figs S4 and S5,

In Fig. 3, we plot systematically the zero frequency value of the real part of the particle-hole pair susceptibility as a function of total momentum *p*, with and without the feedback effect. The curves are computed at a relatively low *T* = 0.3*T*_c_ at unitarity, where *T*_*c*_ is calculated in the absence of the particle-hole channel. Due to the large excitation gap Δ = 0.69*E*_F_, at *p* = 0, the value 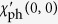 with the feedback is strongly suppressed from its undressed counterpart, 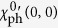. In other words, *the neglect of the self-energy feedback in* 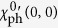 *leads to serious over-estimate of the particle-hole channel contributions*. At the same time, 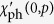 exhibits a more complex, nonmonotonic dependence on *p* than 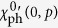. In both cases, the momentum dependence is strong.

Figure 3 and Supplementary Figs S1–S5 reveal that the particle-hole susceptibility 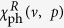 has very strong dependencies on both frequency and momentum, as well as the temperature and interaction strength.

### Induced interaction – beyond the lowest order

Except for the constant factor, 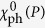, in the absence of the self-energy feedback, is in fact the lowest order induced interaction, considered in GMB^30^ and most others^32^,^33^,^34^,^35^ in the literature:


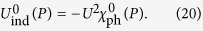


Diagrams of the same order but between fermions of the same spin vanish.

**Figure 4:**
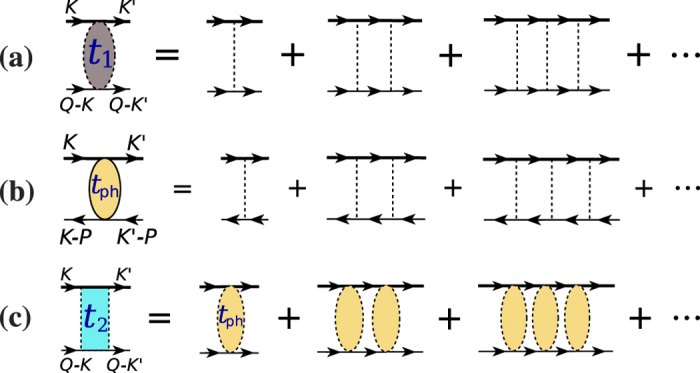
Feynman diagrams showing the particle-hole channel effect on fermion pairing, in the presence of self-energy feedback. (**a**) Particle-particle *T* matrix *t*_1_(*Q*), with external four momenta labeled. (**b**) Particle-hole *T* matrix *t*_ph_(*P*), with *P* = *K* + *K*′ − *Q* being the total particle-hole 4-momentum. (**c**) An effective, composite particle-particle *T*-matrix, *t*_2_(*Q*), with the contribution from the particle-hole channel included. Here different shadings represent different *T* matrices.

Let us first re-plot in Fig. 4(a) the particle-particle scattering *T*-matrix, *t*_pg_, shown in Fig. 1 but now with external legs, referred to as *t*_1_(*Q*). We have


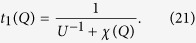


Now we consider the contribution of an infinite particle-hole ladder series, as shown in Fig. 4(b), which should replace the bare interaction *U*. The summation gives rise to the *T*-matrix in the particle-hole channel,


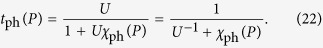


At *Q* = 0, this gives the overall effective pairing interaction,


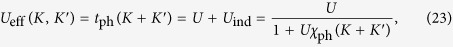


where ±*K* and ±*K*′ are the incoming and outgoing 4-momenta of the scattering particles in the COM reference frame. The induced interaction is thus given by


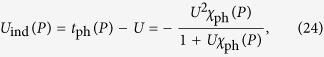


with *P* = *K* + *K*′. Upon Taylor expanding the denominator in powers of *Uχ*_ph_, the leading term, −*U*^2^*χ*_ph_, is the counterpart lowest-order induced interaction in our theory, except that we always consider the self energy feedback effect.

It is evident that the *T* matrices in the particle-particle channel and the particle-hole channel share the same lowest order term, *U*. Both *T* matrices can be regarded as a renormalized interaction, but in different channels. What we need is to replace the bare *U* in one of the two *T* matrices with the other *T* matrix. The results are identical, which we call *t*_2_. Shown in Fig. 4(c) is the regular particle-particle channel *T* matrix *t*_1_(*Q*) with *U* replaced by the particle-hole channel *T* matrix *t*_ph_(*P*) (with twisted external legs), where *P* = *K* + *K*′ − *Q*. In other words, we replace *U*^−1^ with 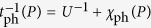 in Eq. (21), and formally obtain


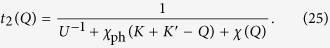


Unfortunately, since *U*_eff_(*K, K*′) is *not* a separable potential, one *cannot* obtain a simple summation in the form of Eq. (25). This can also be seen from the extra dependence on *K* and *K*′ on the right hand side of the equation. Certain averaging process has to be done to arrive at such a simple summation, as will be shown below.

### Gap equation from the self-consistency condition in the mean-field treatment

The dependence of *U*_eff_(*P*) on external momenta via *P* = *K* + *K*′ − *Q* presents a complication in the gap equation. This can be seen through the self consistency condition in the mean field treatment, even though we do not use mean field treatment in our calculations. Writing the interaction *V*_*K*,*K*′_ = *U*_eff_(*K* + *K*′) for *Q* = 0, i.e., zero total pair 4-momentum, we have


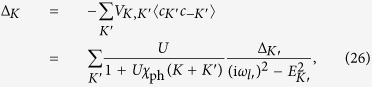


where we have used the mean-field result 〈*c*_*K*′_*c*_−*K*′_〉 = *G*(*K*′)*G*_0_(−*K*′)Δ_*K*′_. Equivalently, this can be written as


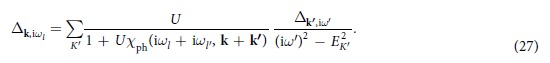


Note that, due to the dynamic character of *χ*_ph_(*K* + *K*′), both the gap Δ_*K*_ and the quasiparticle dispersion *E*_*K*_ acquire a dynamical frequency dependence. The gap also develops a momentum dependence, which is originally absent for a contact potential.

We can express *U*_eff_(*P*) in terms of its retarded analytical continuation, as follows:


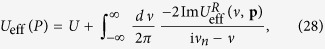


where the second term is just the induced interaction,


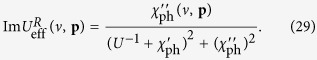


Then we have


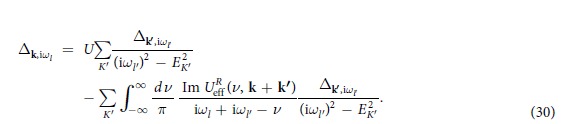


The particle-hole channel effect is contained in the 2nd term, without which this would be just the gap equation without the particle-hole channel, and admit a constant gap solution. Without further approximation, the complex dynamic structure of *χ*_ph_(*P*) will inevitably render it very difficult to solve the gap equation.

### Pairing instability condition in the presence of the particle-hole channel effect

In order to obtain a simple form as Eq. (25), we have to average out the dependence of *U*_eff_(*K, K*′) on *K* and *K*′. Indeed, an average of *χ*_ph_(*ν, p*) has been performed in the literature on (and only on) the Fermi surface^30^. For the frequency part, here we follow the literature and take i*ν*_*n*_ = i*ω*_*l*_ + i*ω*_*l*′_ = 0. From Supplementary Fig. S1, one can see that this is where the imaginary part 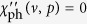 for all *p* and thus the effective interaction *U*_eff_(*K, K*′) is purely real. For the momentum part, we choose on-shell, elastic scattering, i.e., *k* = *k*′, and then average over scattering angles:


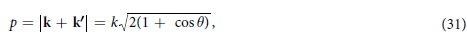


where *θ* is the angle between **k** and **k**′. It is the off-shell scattering processes which lead to imaginary part and nontrivial frequency dependence in 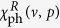 and the order parameter. Further setting *k* = *k*_*μ*_ and averaging only on the Fermi surface is the averaging process used in all papers we can find about induced interactions in the literature. We refer to this as *level 1* averaging. In this paper, we also perform a *level 2* average, over a range of *k* such that the quasiparticle energy *E*_**k**_ ∈ [min(*E*_**k**_), min(*E*_**k**_) + Δ]. Here min(*E*_**k**_) = Δ if *μ* > 0, or 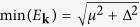 if *μ* < 0. The basic idea is that according to the density of states of a typical *s*-wave superconductor, the states within the energy range *E*_**k**_ ∈ [Δ, 2Δ] are most strongly modified by pairing. It should be pointed out that in the BEC regime, this range can become very large.

Upon averaging of either level 1 or level 2, we drop out the complicated dynamical structure of *χ*_ph_(*ν, p*) and replace it by a constant 〈*χ*_ph_〉. For the purpose of comparison, we shall also perform the averaging on the undressed particle-hole susceptibility 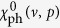 but will mostly show the result at level 1.

**Figure 5:**
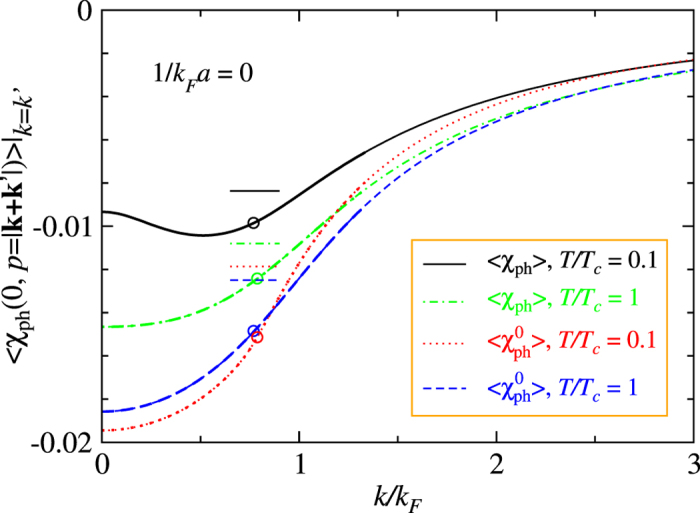
Angular average of the on-shell particle-hole susceptibility, 〈*χ*_ph_(0, *p* = |k + k′|)〉 at *ν* = 0 as a function of momentum *k*/*k*_F_, under the condition *k* = *k*′, calculated at unitarity for different temperatures *T* = 0.1*T*_c_ (black solid curve) and *T* = *T*_c_ (green dot-dashed curve), in units of 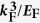. Also plotted is its undressed counterpart, 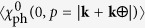, which shows a serious over-estimate due to the neglect of the self-energy feedback. Here *T*_c_ = 0.256*E*_F_ and associated gap and *μ* values are calculated without the particle-hole channel effect. The open circles on each curve denote level 1 average, i.e., *k* = *k*_*μ*_. The vertical axis readings of the horizontal short bars indicate the corresponding values of level 2 average. The thick section of each curve indicates the range of *k* used for level 2 averaging. Clearly, there are strong temperature and *k* dependencies in both 〈*χ*_ph_(0, *p*)〉 and 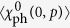. The (absolute) values of Level 2 average are substantially smaller than their level 1 counterpart.

Shown in Fig. 5 are the angular averages of the particle-hole susceptibility at *ν* = 0 as a function of momentum *k* under the above on-shell condition, *k* = *k*′. Here we only show the unitary case at two different temperatures, *T* = *T*_c_ and low *T* = 0.1*T*_c_ ≪ *T*_c_. For the purpose of comparison, we plot the result for both the dressed and undressed particle-hole susceptibility. The curves show strong momentum dependencies. For 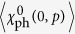, it is monotonically increasing, whereas for 〈*χ*_ph_(0, *p*)〉, it exhibits nonmonotonic *k* dependence at low *T*. Both dressed and undressed particle-hole susceptibilities have a temperature dependence, and this dependence is much stronger for the former. This can be attributed mainly to the temperature dependence of Δ(*T*) in 〈*χ*_ph_(0, *p*)〉, while 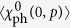 depends on *T* only via *μ*(*T*).

The open circles on each curve represent the level 1 average, i.e., the values at *k* = *k*_*μ*_. At the same time, the vertical axis readings of the short horizontal bars correspond to the level 2 average, while the thick segments of each curve represents the range of *k* used for level 2 averaging. Figure 5 shows that the (absolute) values of the level 2 average are significantly smaller than their level 1 counterpart. The level 1 average 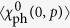 is essentially temperature independent (see the red and blue circles). In addition, it is evident that *the neglect of self-energy feedback has caused* 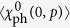 *to seriously over-estimate the contribution of particle-hole channel.*

Similar plot for 1/*k*_F_*a* = 0.5 (Supplementary Fig. S6) exhibits a much stronger *T* dependence. In that case, *μ* is very close to 0 albeit still positive. As a consequence, the particle-hole susceptibility is much smaller than that shown in Fig. 5.

Now with this frequency and momentum independent *χ*_ph_(*ν, p*) ≈ 〈*χ*_ph_〉, we can easily carry out the simple geometric summation for *t*_2_:


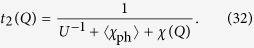


Therefore, the Thouless criterion for pairing instability leads to the gap equation:


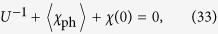


namely,


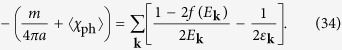


As will be shown later, 〈*χ*_ph_〉 is always negative. Therefore, the particle-hole channel effectively reduces the strength of the pairing interaction.

In the weak coupling limit (1/*k*_F_*a* = −∞), Δ → 0, 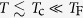, then 〈*χ*_ph_〉 and 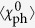 become equal, for either level of averaging. We have


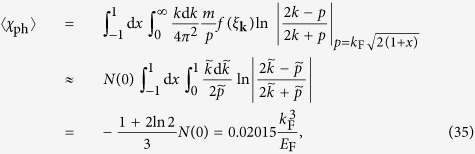


where 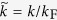, 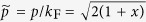, *x* = cos *θ*, and *N*(0) = *mk*_F_/2*π*^2^*ħ*^2^ is the density of state at the Fermi level. Here we have approximated the Fermi function with its *T* = 0 counterpart, with a step function jump at the Fermi level.

In the weak interaction limit, the BCS result for *T*_c_ is 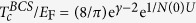, where *γ* ≈ 0.5772157 is the Euler’s constant. Equation (33) implies a replacement of 1/*U* by 1/*U* + 〈*χ*_ph_〉. In this way, the new transition temperature *T*_c_ is given by


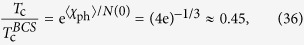


and the same relation holds for zero *T* gap,


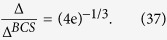


This result is in *quantitative* agreement (to the leading order) with that of GMB^30^ and others^32^ in the literature. Note that in our work, as well as in that of Yin and coworkers^35^, the average particle-hole susceptibility 〈*χ*_ph_〉 is added to 1/*U* or *m*/4*πa*. In other works^32^,^33^,^34^, only the lowest order particle-hole diagram is considered so that their induced interaction 
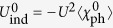
 is added to *U*. Therefore, these works have to rely on the assumption *N*(0)*U* ≪ 1 and the validity of the BCS mean-field result in order to obtain the result of Eq. (36). Away from the weak interaction regime, a full summation of the particle-hole *T* matrix becomes necessary.

While the results from all different treatment seem to agree quantitatively in the weak coupling limit, we expect to see significant departures as the pairing interaction strength increases, especially in the unitary regime.

With the overall effective interaction *U*_eff_, the self energy, as obtained from 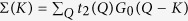, will follow the same form as Eq. (8) although the gap values will be different. Therefore, the fermion number equation will also take the same form as Eq. (10). Furthermore, the pseudogap equation, given by 
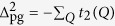
, will also take the same form as Eq. (7).

Equations (10), (7), and (34) now form a new closed set, and will be solved to investigate the effect of the particle-hole channel.

Note that in a *very dilute* Fermi gas shifting *m*/4*πa* by 〈*χ*_ph_〉 has no significant influence in experimental measurement of the *s*-wave scattering length *a*, because 〈*χ*_ph_〉 has dimension [*k*_F_]^3^/[*E*_F_] = [*k*_F_] and thus vanishes as *k*_F_ → 0 in the zero density limit. However, a finite *k*_F_ will indeed shift the resonance location except at very high *T* where *μ* < 0. In ref. 58, from which the scattering lengths are often quoted for ^6^Li, the actual density is comparable or even higher than that in most typical Fermi gas experiments. Therefore, the particle-hole channel may play an important role.

*Here we propose that this particle-hole channel effect may be verified experimentally* by precision measurement of the magnetic field *B* at the exact Feshbach resonance point as a function of density or *k*_F_ at low *T*. The zero density field *B*_0_ can be obtained by extrapolation. Then one should have the field detuning *δB* = *B*−*B*_0_ ∝ *k*_F_. Because different theories predict a very different value of 〈*χ*_ph_〉 at unitarity, the measured field detuning can thus be used to quantify 〈*χ*_ph_〉 and test these theories. In principle, one may experimentally measure 〈*χ*_ph_〉 through the entire BCS-BEC crossover. For a Fermi gas in a trap, the trap inhomogeneity leads to a distribution of *k*_F_. Instead of a uniform shift, this inhomogeneity will spread out the unitary point at zero density into a narrow band at finite density. The band width and mean shift are both expected to be proportional to *k*_F_. Such effect deserves further investigation.

## Numerical Results and Discussions

### Effect of the particle-hole channel on BCS-BEC crossover

In this section, we will investigate the effect of the particle-hole channel on the BCS-BEC crossover behavior, in terms of zero temperature gap Δ(0), *T*_c_ and their ratio.

**Figure 6:**
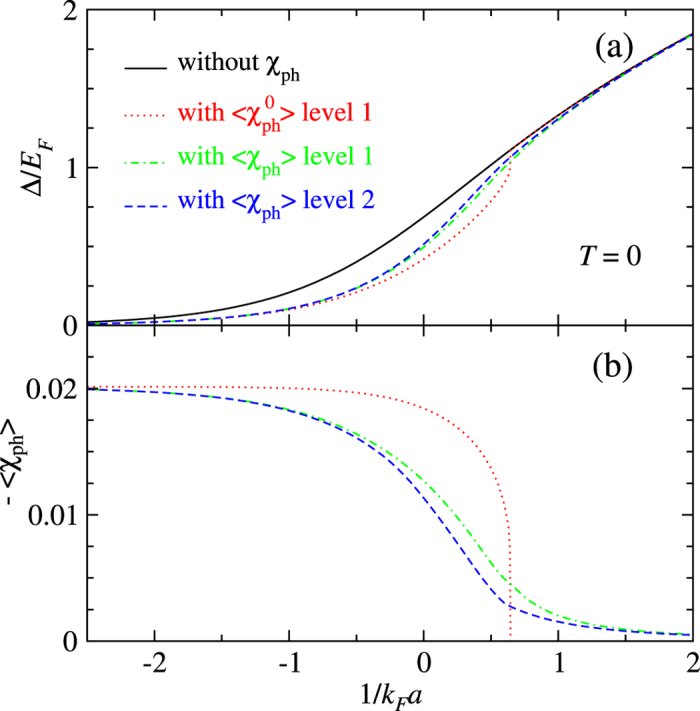
Effect of the particle-hole channel contributions on the zero temperature gap in BCS-BEC crossover. In (**a**), the black solid curve is the gap without the particle-hole effect. The rest curves are calculated with the particle-hole channel effect but at different levels, i.e., using undressed particle-hole susceptibility 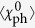 with level 1 averaging (red dotted line), dressed particle-hole susceptibility 〈*χ*_ph_〉 with level 1 (green dot-dashed curve) and level 2 (blue dashed line) averaging, respectively. The corresponding values of the average particle-hole susceptibility with a minus sign are plotted in (**b**), in units of 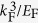. The particle-hole channel effect can be essentially neglected beyond 1/*k*_F_*a* > 1.5.

First, in Fig. 6, we show the effect on the zero *T* gap by comparing the calculated result with and without the particle-hole channel contributions. Shown respectively in panel (a) and (b) are plots of the zero *T* gap Δ and the corresponding particle-hole susceptibility (with a minus sign) as a function of 1/*k*_F_*a*. The black solid line in Fig. 6(a) is the result without the particle-hole channel effect, whereas the other curves are calculated with the effect at different levels of approximation. The (red) dotted curve are calculated using the undressed susceptibility 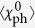 at average level 1. The (green) dot-dashed and (blue) dashed curves are calculated using the dressed particle-hole susceptibility 〈*χ*_ph_〉 with level 1 (green dot-dashed curve) and level 2 (blue dashed line) averaging, respectively. The level 2 result shows a slightly weaker particle-hole channel effect, as can be expected from Fig. 5.

One feature that is easy to spot is that the undressed particle-hole susceptibility 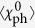 has a very abrupt shut-off where the chemical potential *μ* changes sign. As a result, the corresponding (red dotted) curve of the gap also merges abruptly with the (black solid) gap curve calculated without particle-hole channel effect. This is *not unexpected* as one can see from Eq. (19) that 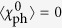 at *T* = 0 for *μ* ≤ 0. Furthermore, Eq. (18) implies that 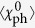 approaches zero at *μ* = 0 abruptly with a finite slope as *k*_*μ*_ does. In contrast, with the self-energy feedback included, either level 1 (green dot-dashed curves) or level 2 (blue dashed curves) average of 〈*χ*_ph_〉 approaches 0 smoothly as the BEC regime is reached. Consequently, in Fig. 6(a), the (green) dot-dashed and (blue) dashed curves approach the (black) solid curve very gradually. It is also worth pointing out that the difference between level 1 and level 2 average of 〈*χ*_ph_〉 is less dramatic than that between 〈*χ*_ph_〉 and the undressed 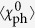. Indeed, the (green) dot-dashed and (blue) dashed curves are very close to each other. The abrupt shut-off of 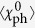 at *μ* = 0 is determined by the step function characteristic of the Fermi function at *T* = 0.

In the unitary regime, especially for 1/*k*_F_*a* ∈ [−0.5, +0.5], the particle-hole susceptibility is strongly over-estimated by the undressed 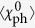 in comparison with the dressed 〈*χ*_ph_〉. In this regime, both the gap and the underlying Fermi surface (as defined by the chemical potential) are large, so that neglecting the self-energy feedback leads to a strong over-estimate of 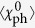, because the large gap serves to suppress particle-hole fluctuations.

From Fig. 6, we conclude that the particle-hole effect diminishes quickly as the Fermi gas is tuned into the BEC regime with increasing pairing interaction strength. Beyond 1/*k*_F_*a* > 1.5, the effect can essentially be neglected. For the level 1 average of the undressed particle-hole susceptibility, 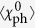, as has been done in the literature, this effect disappears immediately once the BEC regime (defined by *μ* < 0) is reached, as far as the zero *T* gap is concerned.

As a consistency check, we notice that in the BCS limit, the average particle-hole susceptibility in all cases in Fig. 6(b) approaches the same asymptote, which is given by Eq. (35). This confirms our previous analytical analysis.

**Figure 7:**
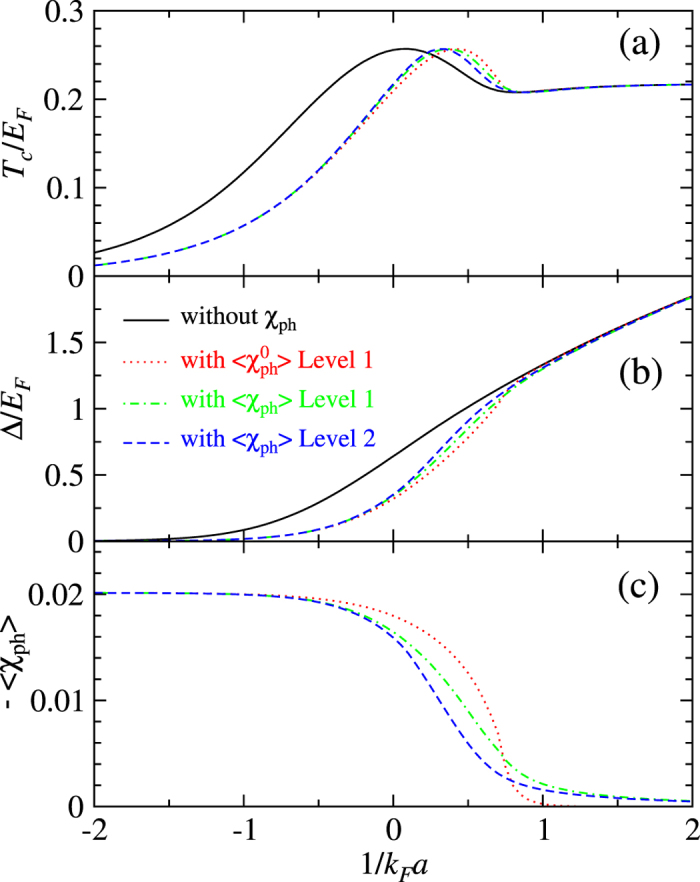
Effect of the particle-hole channel contributions on *T*_c_ and the pseudogap Δ at *T*_c_ in BCS-BEC crossover. In (**a,b**), the black solid curves are calculated without the particle-hole effect. The rest curves are calculated with the particle-hole channel effect but at different levels, using undressed particle-hole susceptibility 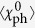 with level 1 averaging (red dotted line), dressed particle-hole susceptibility 〈*χ*_ph_〉 with level 1 (green dot-dashed curve) and level 2 (blue dashed line) averaging, respectively. The corresponding values of the average particle-hole susceptibility with a minus sign are plotted in (**c**), in units of 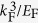. The particle-hole channel effect can be essentially neglected beyond 1/*k*_F_*a* > 1.5.

Next, we show in Fig. 7 the effect of the particle-hole channel on the behavior of *T*_c_ as well as the pseudogap at *T*_c_. Figure 7(c) can be compared with Fig. 6(b). The curves for levels 1 and 2 average of 〈*χ*_ph_〉 in Fig. 7(c) are very similar to those in Fig. 6(b), with the values at 1/*k*_F_*a* = 0 slightly smaller. On the other hand, the curve for 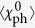 has a smooth thermal exponential tail in the BEC regime in Fig. 7(c). Thus, the pseudogap Δ(*T*_c_) calculated using 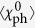 now merges back to the (black) solid curve smoothly.

Similar to the zero *T* gap case in Fig. 6, the difference in the effect on *T*_c_ and Δ(*T*_c_) between level 1 and level 2 averaging mainly resides in the unitary regime, and is less dramatic than that between undressed and dressed particle-hole susceptibility. Again, *the undressed particle-hole susceptibility gives rise to an overestimate of the particle-hole channel effect.*

In all cases, the particle-hole susceptibility becomes negligible in the BEC regime. The effect of the particle-hole channel shifts the *T*_c_ and Δ(*T*_c_) curve towards larger 1/*k*_F_*a*, although the amount of shift clearly depends on the value of 1/*k*_F_*a*.

Now we study the effect of the particle-hole channel on the ratio 2Δ(0)/*T*_c_. It suffices to consider the mean-field ratio, 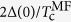, since 2Δ(0)/*T*_c_ obviously will deviate from the weak coupling BCS result when pairing fluctuations are included in the crossover and BEC regimes. From Fig. 5, we see a strong *T* dependence of the particle-hole susceptibility. Therefore, the effect on 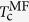 and on zero *T* gap Δ(0) are different, as can be seen roughly from Figs 6 and 7.

**Figure 8:**
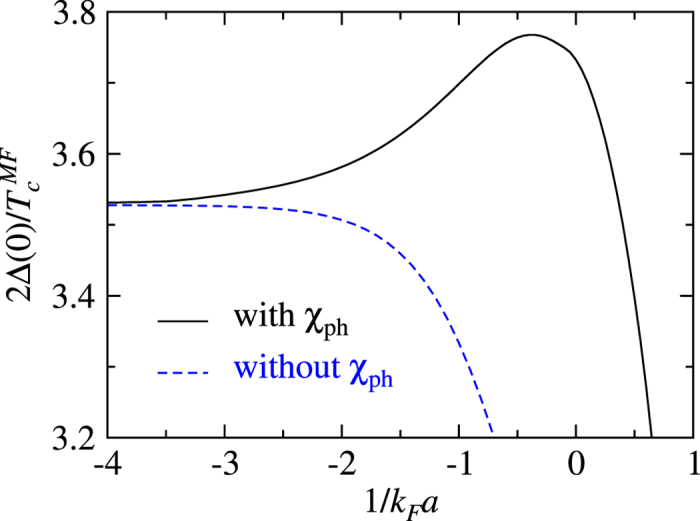
Effect of the particle-hole channel contributions on the ratio 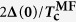 in BCS-BEC crossover. Shown is the mean-field ratio calculated with (black solid curve) and without (blue dashed curve) the particle-hole channel contributions. Here the particle-hole susceptibility 〈*χ*_ph_〉 is calculated with level 2 averaging.

In Fig. 8, we plot this mean-field ratio as a function of 1/*k*_F_*a* with (black solid curve) and without (blue dashed curve) the particle-hole channel effect. Here the particle-hole susceptibility 〈*χ*_ph_〉 is calculated with level 2 averaging. In the 1/*k*_F_*a* → −∞ limit, the ratio is unaffected by the particle-hole channel. As 1/*k*_F_*a* increases, the contribution of the particle-hole channel causes this ratio to increase gradually. At 1/*k*_F_*a* = −4, which is still a very weak pairing case, the ratio is already slightly larger. The effect is most dramatic in the unitary regime, since further into the BEC regime, 〈*χ*_ph_〉 will vanish gradually. It is worth noting that even without the particle-hole channel, the ratio 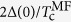 starts to decrease from its weak coupling limit, 2*π*e^−*γ*^ ≈ 3.53.

Finally, we estimate the shift in Feshbach resonance positions. From Figs 6 and 7, we find that *χ*_ph_ does not necessarily diminish as *T* increases except at very high *T* (where *μ* becomes negative, so that |*χ*_ph_| will decrease exponentially with *T*.) In fact, this can be understood because Δ(*T*) decreases with *T* so that |*χ*_ph_| increases. We take 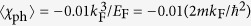. According to Eq. (34), the shift in 1/*a* is *δ*(1/*a*) = −4*πħ*^2^〈*χ*_ph_〉/2*m* = 0.08*πk*_F_. In other words, the dimensionless shift *δ*(1/*k*_F_*a*) = 0.25, which is independent of density and is no longer negligible. This is in good agreement with the actual shift 0.32 of the peak location of the *T*_c_ curve in Fig. 7(a). For a typical *T*_F_ = 1 *μ*K in ^6^Li, using the approximate expression *a* = *a*_bg_[1 − *W*/(*B* − *B*_0_)], we obtain the shift in resonance position *δB*_0_ = −0.08*W*(*k*_F_*a*_bg_) = 7.8 G. Here for the lowest two hyperfine states, the resonance position *B*_0_ = 834.15 G, the resonance width *W* = 300 G, and the background scattering length *a*_bg_ = −1405*a*_0_, with *a*_0_ = 0.528 Å. Clearly, the shift *δB*_0_ is not small. In reality, one needs to solve self-consistently the equation *m*/(4*πa*) + 〈*χ*_ph_〉 = 0, and take care of the trap inhomogeneity. These will likely make the actual average shift smaller.

The susceptibility *χ*_ph_ calculated with and without the self energy feedback differs by roughly a factor of 2 at unitarity. This can be used to test different theories, as mentioned earlier.

A question arises naturally as to whether the particle-hole channel effect has already been included in the experimentally measured scattering length *a*, since, after all, the measurements of *a* such as those in ref. 58 were carried out at densities comparable to typical Fermi gas experiments. This also depends on whether the temperature was high enough during the measurements.

### Critical temperature *T*_c_ at unitarity

Finally, we compare our result on the critical superfluid transition temperature *T*_c_/*E*_F_ for a 3D homogeneous Fermi gas at unitarity with those reported in the literature. From Fig. 7, we read *T*_c_/*E*_F_ = 0.217 using level 2 average of 〈*χ*_ph_〉. And the maximum *T*_c_ ≈ 0.257 now occurs at 1/*k*_F_*a* ≈ 0.32, on the BEC side. The level 1 average of 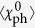 yields a slightly lower value, *T*_c_/*E*_F_ = 0.209. However, we emphasize that the level 2 average of 〈*χ*_ph_〉 is more reasonable. Note that as in the theory without particle-hole channel effect, we have dropped out the incoherent part of the self-energy from the particle-particle scattering. Inclusion of the incoherent part is necessary in order to obtain the correct value of the *β* factor.

Hu and coauthors^59^,^60^ have been claiming to be able to obtain the correct value of the *β* factor, using an NSR-based approach, *without including the particle-hole channel*. We note that this claim will breakdown when the particle-hole channel is included.

The value of *T*_c_ for a homogeneous Fermi gas at unitarity has been under intensive study over the past few years, both theoretically and experimentally, or using Monte Carlo simulations. The theory results for *T*_*c*_/*T*_*F*_ range from 0.13^61^ to 0.264^62^. Various experiments report a large range as well, with a recent value of 0.167^51^. We emphasize that, given the poor precision in experimental measurements, these measured values are far from being conclusive. More detailed comparison can be found in ref. 52.

Including the particle-hole channel contributions has reduced substantially our value of *T*_*c*_, bringing it closer to the most recent experimental data. We expect that including the incoherent part of the self energy (*δ*Σ) in Eq. (6) should lower the chemical potential and thus reduce *T*_*c*_ further. Indeed, if we take an constant *δ*Σ = −0.3*E*_F_ (half of the energy of a single spin down atom in a spin up Fermi sea^63^), *T*_c_/*E*_F_ will be suppressed down to 0.174, close to the recent experimental value. Full numerical inclusion of *δ*Σ will be done in a future study.

### Higher order corrections

**Figure 9:**
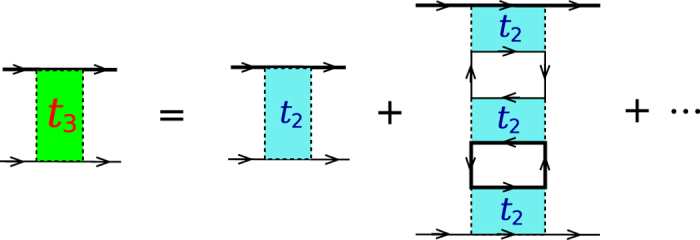
Higher order *T*-matrix, *t*_3_, obtained by repeating the *T*-matrix *t*_2_.

In addition to non-ladder diagrams, which we have chosen not to consider, there seem to be a series of higher order corrections. For example, one can imagine repeating the *T*-matrix *t*_2_ in the way shown in Fig. 9, and obtaining a higher order *T*-matrix *t*_3_. Such *t*_3_ can then be repeated to obtain a higher order *T*-matrix *t*_4_, and so on. While one may argue these higher order *T*-matrices are indeed of higher order in bare interaction *U*, our experience with *t*_2_ seems to imply that detailed study needs to be carried out before we jump to a conclusion on this. Indeed, even the lowest order so-called induced interaction 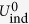 is one order higher in *U* than *U* itself.

### Note added:

Our manuscript was initially posted at arXiv (arXiv:1109.2307). Since then, there have been new results from QMC on the zero temperature ground state energy of a unitary Fermi gas^50^. We have also learned of the QMC result from Ref. 49. Both are in good agreement with experimental results in Ref. 51.

## Conclusions

In summary, we have studied the effects of the particle-hole channel on BCS-BEC crossover and compared with lower level approximations. We include the self-energy feedback in the particle-hole susceptibility *χ*_ph_, which leads to substantial differences than the result without self-energy feedback.

We have investigated the dynamic structure of *χ*_ph_, and have discovered very strong temperature, momentum and frequency dependencies. Angular (as well as radial) average in the momentum space of the particle-hole susceptibility has been done in order to keep the equations manageable. We have performed the average at two different levels and also compared with the result calculated without including the self-energy feedback. We conclude that the level 2 averaging, i.e., both over angles and a range of momentum, is more reasonable. Computations of the particle-hole susceptibility without the self-energy feedback leads to an overestimate of the particle-hole channel effect.

In the weak coupling BCS limit, our result agrees, to the leading order, with that of GMB and others in the literature. Away from the weak coupling limit, Δ(0) and *T*_c_ are suppressed differently. We have also studied the ratio 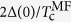 at the mean-field level and found that it is modified by the particle-hole fluctuations. The particle-hole channel effects diminish quickly once the system enters the BEC regime.

Since the particle-hole channel effectively renormalizes the pairing strength, therefore, it is important to have the particle-hole channel properly addressed, in order to make quantitative comparisons with experiment. This suggests that many theoretical calculations in the literature deserve to be revisited.

Without including the incoherent part of the self energy from particle-particle scattering, our present result on the critical temperature at unitarity yields *T*_c_/*E*_F_ ≈ 0.217, substantially lower than that obtained without the particle-hole effect. This value agrees reasonably well with some existing experimental measurement.

We have also made a falsifiable proposal that the particle-hole contribution can be measured by locating the Feshbach resonance positions as a function of *k*_F_ and that this can be used to test different theories.

To study more accurately the quantitative consequences of the dynamic structure of the particle-hole susceptibility, full-fledged numerical calculations are needed, without taking simple angular average and setting frequency *ν* = 0. Further investigation is called for in order to determine whether higher order *T*-matrices will make a significant difference or not.

## Additional Information

**How to cite this article**: Chen, Q. Effect of the particle-hole channel on BCS–Bose-Einstein condensation crossover in atomic Fermi gases. *Sci. Rep.* **6**, 25772; doi: 10.1038/srep25772 (2016).

## Supplementary Material

Supplementary Information
