# Supplementary material for: Effect of the particle-hole channel on BCS–Bose-Einstein condensation crossover in atomic Fermi gases
Source: Sci Rep. 2016 May 17;6:25772. doi: 10.1038/srep25772 (PMC4868972; doi:10.1038/srep25772)
Supplement: Supplementary Information [file srep25772-s1.pdf]

Supplementary Information

Effect of the particle-hole channel on BCS–Bose-Einstein condensation crossover in atomic Fermi gases

Qijin Chen
Department of Physics and Zhejiang Institute of Modern Physics,
Zhejiang University, Hangzhou, Zhejiang 310027, CHINA
Email: qchen@zju.edu.cn

(Dated: February 15, 2016)

Abstract

Here we present more details of the complex dynamic structure of the particle-hole susceptibility $\chi_{\rm ph}$, and analyze its evolution with temperature, pairing strength, and particle-hole momentum. Where appropriate, we compare with the undressed particle-hole susceptibility $\chi^0_{\rm ph}$. Finally, we present an angular average of $\chi_{\rm ph}$ in the BEC regime.

I. DYNAMIC STRUCTURE OF THE PARTICLE-HOLE SUSCEPTIBILITY: 3D PLOTS

Here we show in the supplementary materials the Dynamic structure of the particle-hole susceptibility, with and without the self-energy feedback.

Shown in Figs. S1(a) and S1(b) are three dimensional (3D) plots of the real and imaginary parts of $\chi^{0R}_{\text{ph}}(\nu, \mathbf{p})$. In Figs. S1(c) through S1(f) we present our calculated results of $\chi^{R}_{\text{ph}}(\nu, \mathbf{p})$ in the presence of self energy feedback. They are calculated at T_c (a-d) and $0.1T_c$ (e,f) in the unitary limit, $1/k_Fa = 0$. Here $T_c/E_F \approx 0.256$ is the one calculated in the pairing fluctuation theory without including the particle-hole channel contribution.

The even and odd symmetries of $\chi^{0\prime}_{\text{ph}}(\nu, \mathbf{p})$ and $\chi^{0\prime\prime}_{\text{ph}}(\nu, \mathbf{p})$ with respect to $\nu \to -\nu$ are evident. And indeed these symmetries are not present for $\chi^{R}_{\text{ph}}(\nu, \mathbf{p})$.

The interesting structure in Figs. S1(c) through S1(f) at low frequency and low momentum clearly derives from the pseudogap already present at T_c in the fully dressed Green's function. In other words, by neglecting the feedback effect, the bare $\chi^{0}_{\text{ph}}(P)$ misses this important dynamic structure. The peaks in the plots of χ^{0R}_{ph}, as shown in Figs. S1(a) and S1(b), would become sharper as the temperature decreases.

Comparing Figs. S1(e) and S1(f) with S1(c) and S1(d), the gap induced structures become much more pronounced. For example, for $p = 0$, the range of ν in which $\chi''_{\text{ph}}(\nu, 0) = 0$ becomes much wider at low T.

II. DYNAMIC STRUCTURE OF THE PARTICLE-HOLE SUSCEPTIBILITY: 2D PLOTS

Next, we will present a series of two-dimensional plots, in order to make the 3D data shown in Fig. S1 quantitatively easier to read. Then we study in detail the effect of temperature and interaction strength on the particle-hole susceptibility, and how it behaves as a function of frequency ν for fixed momentum p or as a function of total momentum p for fixed frequency ν.

A. Effect of temperature

First, we study the impact of temperature. We present in Fig. S2 the real and imaginary parts of $\chi^{R}_{\text{ph}}(\nu, 0)$ for different T from low to high in the unitary limit, $1/k_Fa = 0$. To single out the temperature effect, here we take for all temperature $\Delta = 0.686$ and $\mu = 0.59$, which are their values calculated at $T = 0$ using the pairing fluctuation theory without the particle-hole channel

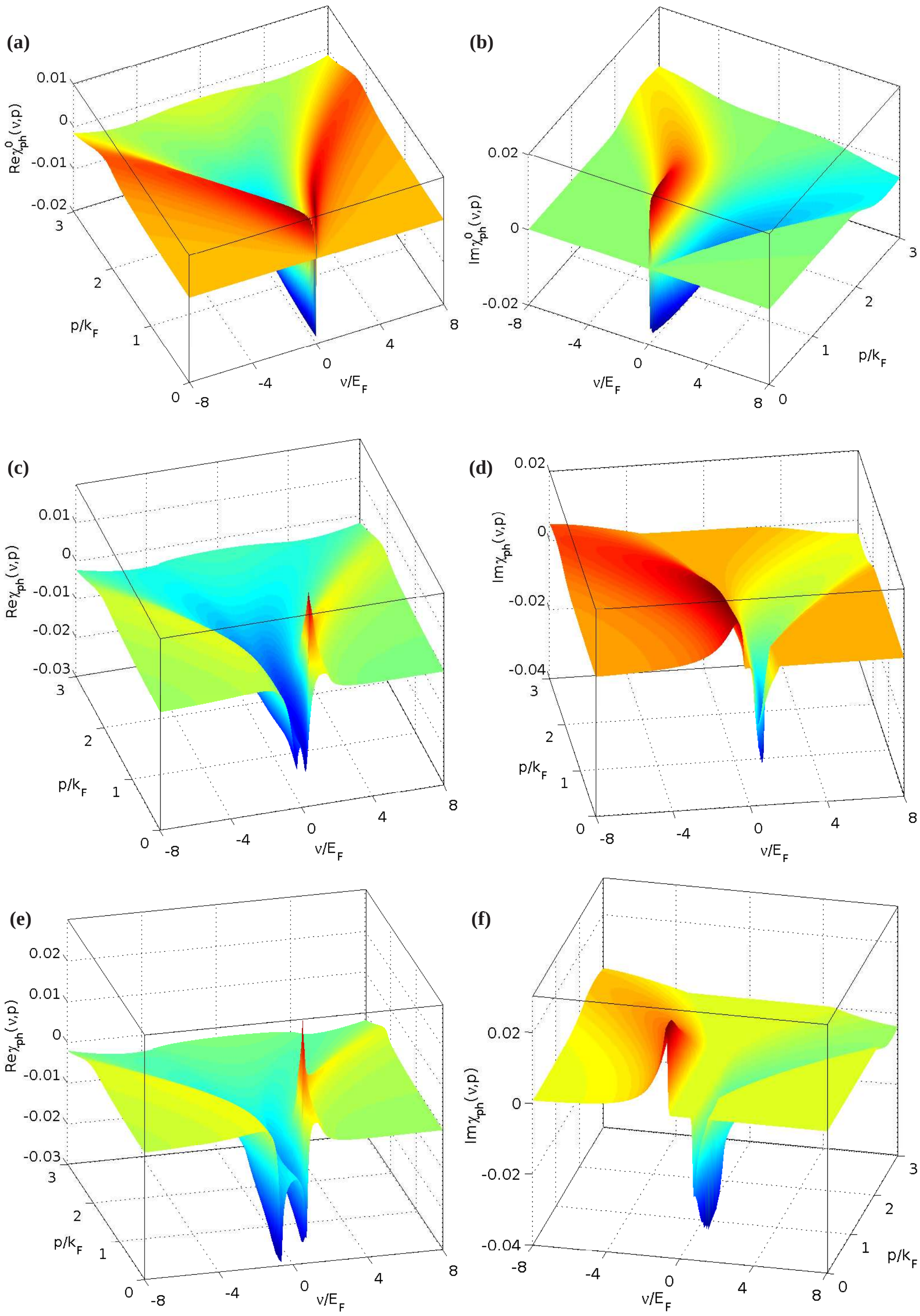

Figure S1. 3D plots of the real (a,c,e) and imaginary (b,d,f) parts of the particle-hole pair susceptibility $\chi_{\rm ph}$ with (c-f) and without (a,b) the self-energy feedback, calculated at $T_{\rm c}$ (a-d) and $0.1T_{\rm c}$ (e,f) in the unitary limit. Here $T_{\rm c}$ is calculated using the pairing fluctuation theory without the particle-hole channel contributions. While the undressed $\chi_{\rm ph}^{0R}(\nu, p)$ has a simple symmetry under $\nu \rightarrow -\nu$, the dynamic structure of $\chi_{\rm ph}^{R}(\nu, p)$ is much more complex. In both cases, the real and imaginary parts have very strong dependencies on the total frequency ν and total momentum p. $\chi_{\rm ph}^{R}(\nu, p)$ shows strong gap effects both at $T_{\rm c}$ and low T. In units of $E_{\rm F}$, the parameters are: $T_{\rm c} = 0.256$, $\mu(T_{\rm c}) = 0.62$, $\Delta(T_{\rm c}) = 0.64$, $\mu(0.1T_{\rm c}) = 0.59$, and $\Delta(0.1T_{\rm c}) = 0.69$.

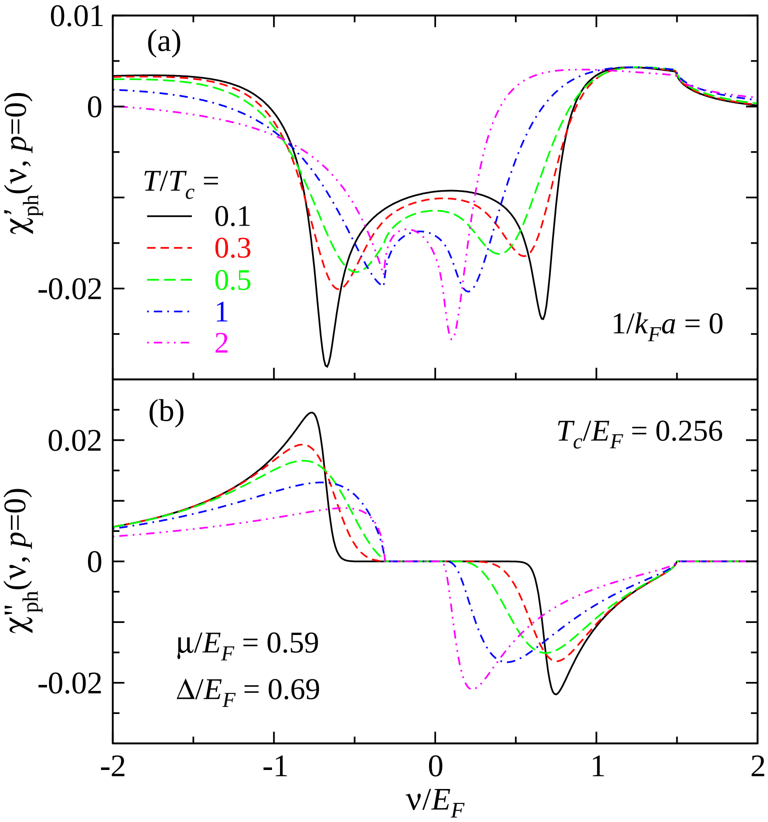

Figure S2. The particle-hole susceptibility $\chi^R_{\rm ph}(\nu, 0)$ at zero momentum p in the presence of self-energy feedback for different T (as labeled) at unitarity. To single out the temperature effect, we fix $\Delta = 0.686$ and $\mu = 0.59$ at their values at $T = 0$, calculated using the pairing fluctuation theory without the particle-hole channel effect.

effect. Evidently, beyond the point $\nu = \sqrt{\mu^2 + \Delta^2} + \mu = 1.49$, the imaginary part $\chi''_{\rm ph}(\nu, 0)$ vanishes identically. Around $\nu = 0$, the lower bound of the range of ν where $\chi''_{\rm ph}(\nu, 0)$ essentially vanishes changes from $\nu = -\Delta = -0.686$ at low T to $\nu = -(\sqrt{\mu^2 + \Delta^2} - \mu) = 0.315$ at high T. Meanwhile, its upper bound decreases continuously with T from $\nu = \Delta$ at low T to $\nu = 0$ at very high T. This numerical result agrees with our previous analysis. A comparison with the real part reveals that the peaks in $\chi'_{\rm ph}(\nu, 0)$ correspond to the sharp rises in the plot of $\chi''_{\rm ph}(\nu, 0)$ near these lower and upper bounds. This can also be seen from the Kramers-Kronig relation between the real and imaginary parts of $\chi^R_{\rm ph}(\nu, 0)$.

In comparison, we have also studied the temperature evolution of the undressed $\chi^{0R}_{\rm ph}(\nu, p)$. Shown in Fig. S3 is the result for $p = 0.1$. From Fig. S1, it is easy to see that one cannot plot the result for $p = 0$. This can also be seen from Eqs. (17) and (18) in the main text. For finite p, say $p = 0.1$, the peaks at low T in both real and imaginary parts become more smeared out as T increases. Near $\nu = 0$, we see that $\chi^{0\prime\prime}_{\rm ph}(\nu, p = 0.1)$ is proportional to ν, in agreement with our

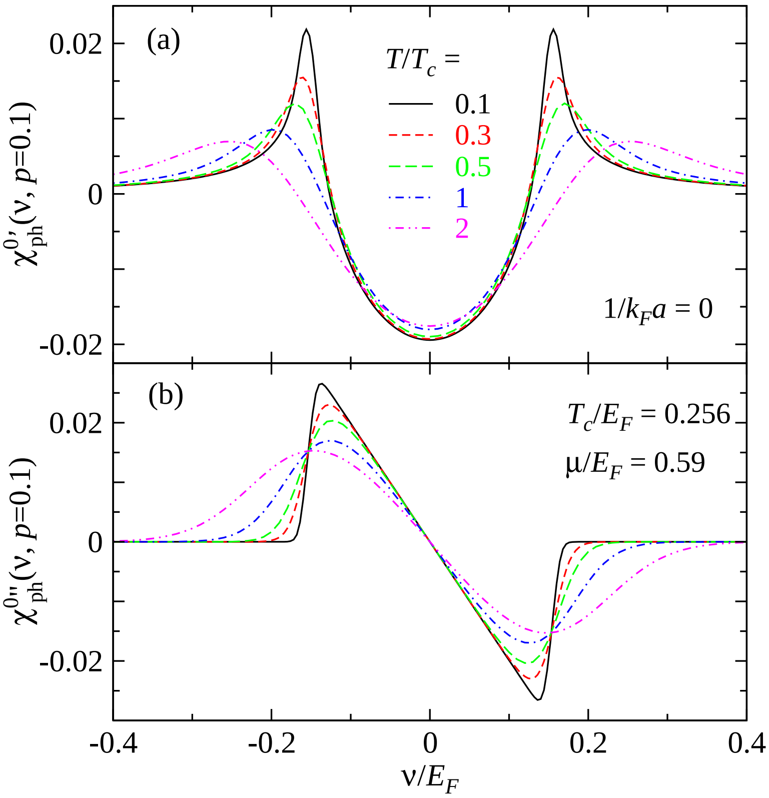

Figure S3. The undressed particle-hole susceptibility $\chi^{0R}_{\rm ph}(\nu, p = 0.1)$ for different T (as labeled) at unitarity and momentum $p = 0.1$. As in Fig. S2, we fix $\mu = 0.59$.

previous analysis. $\chi^{0R}_{\rm ph}(\nu, p)$ shows good symmetry about ν: $\chi^{0R}_{\rm ph}(-\nu, p) = \chi^{0R*}_{\rm ph}(\nu, p)$. There is no gap effects, of course.

B. Evolution of $\chi^{R}_{\rm ph}(\nu, 0)$ with increasing pairing strength.

Shown in Fig. S4 is the evolution of the particle-hole susceptibility $\chi^{R}_{\rm ph}(\nu, 0)$ at total momentum $p = 0$ in the presence of feedback effect with increasing pairing strength. These curves are calculated at low temperature $T = 0.1T_{\rm c}$. Here for each interaction strength, the parameters Δ, μ and $T_{\rm c}$ are calculated using the pairing fluctuation theory without the particle-hole channel effect. Around $\nu = 0$, the range within which the imaginary part vanishes is given by $|\nu| < \Delta$ for the $\mu > 0$ cases ($1/k_{\rm F}a = -1$ through 0.5). For $1/k_{\rm F}a = 1$, $\mu/E_{\rm F} = -0.8$, the lower bound is given by $-(\sqrt{\mu^2 + \Delta^2} - \mu) = -2.35$ and its upper bound extends to ∞ since $\Delta = 1.33 > \sqrt{\mu^2 + \Delta^2} + \mu = 0.75$. It is obvious that this range becomes wider and wider with increasing pairing strength from BCS to BEC.

From Figs. S1(a) and S1(b), one readily notice that for the undressed $\chi^{0R}_{\rm ph}(\nu, p)$, it is not appro-

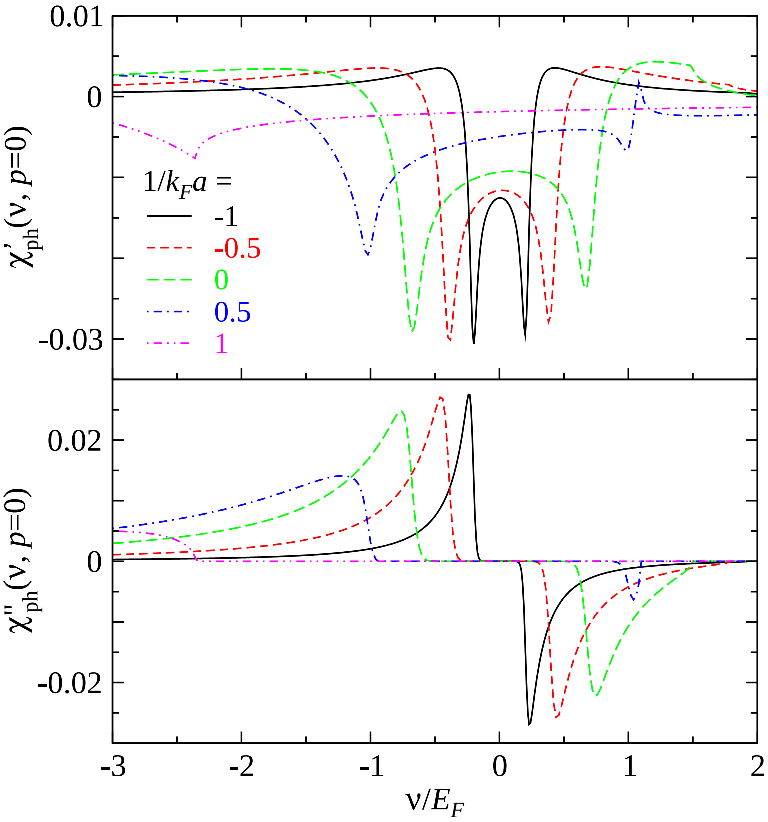

Figure S4. The real and imaginary parts of particle-hole susceptibility $\chi^R_{\text{ph}}(\nu, 0)$ in the presence of self-energy feedback for various values of $1/k_\text{F}a$ from BCS to BEC. The curves are calculated at $0.1T_\text{c}$. For each case, the parameters Δ, μ and T_c are calculated using the pairing fluctuation theory without the particle-hole channel effect.

priate to plot $\chi^{0R}_{\text{ph}}(\nu, p = 0)$ as a function of ν. Instead, one has to plot at a finite p, say, $p = 0.1k_\text{F}$, in order to study its temperature evolution. Our result (not shown) demonstrates that the real part presents a double peak structure, with the peaks becoming increasingly broader as T increases. At low T, the location of the peaks are roughly given by $\nu = \pm pk_\mu/m \approx \pm 0.15$ for $\mu = 0.59$ at unitarity. This relation also shows how the $\chi^{0R}_{\text{ph}}(\nu, p)$ curves evolve with total momentum p.

C. Evolution of $\chi^R_{\text{ph}}(\nu, p)$ with particle-hole momentum p

Next, we investigate how the particle-hole susceptibility $\chi^R_{\text{ph}}(\nu, p)$ evolves with total momentum p in the presence of feedback effect. Shown in Fig. S5 are the curves of the real and imaginary parts for increasing p for a unitary Fermi gas, calculated at T_c. Just as in Fig. S2, the $p = 0$ curve shows a clear gap in the neighborhood of $\nu = 0$ in the imaginary part, $\chi''_{\text{ph}}(\nu, p)$. As p increases, this gap gradually disappears, and the upper bound in ν beyond which $\chi''_{\text{ph}}(\nu, p)$ vanishes increases

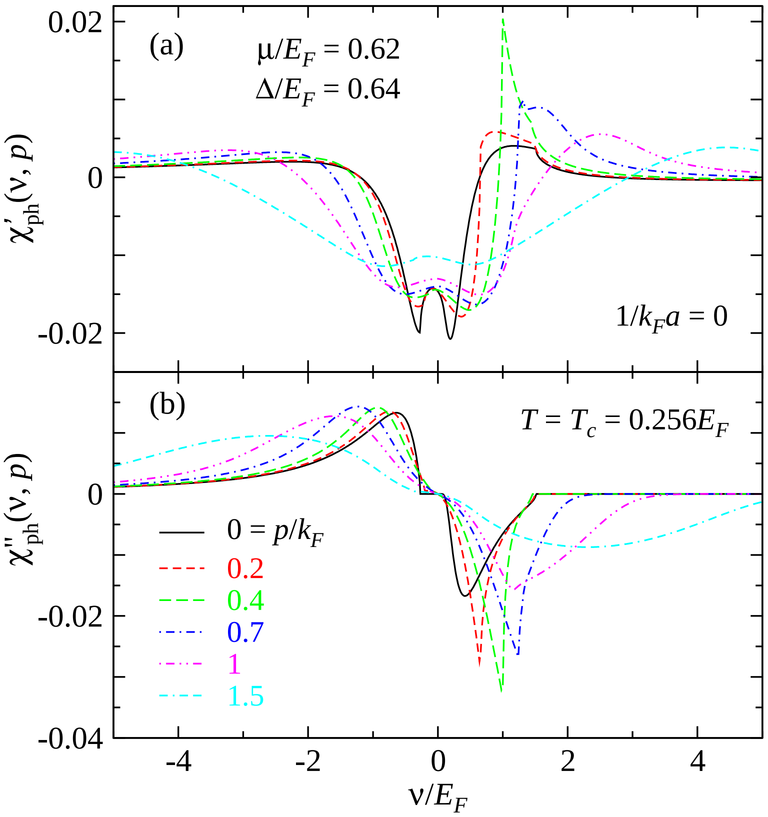

Figure S5. The particle-hole pair susceptibility $\chi^R_{\text{ph}}(\nu, p)$ at unitarity and at T_c in the presence of self-energy feedback for increasing $p = 0$, 0.2, 0.4, 0.7, 1, and 1.5. The parameters Δ, μ and T_c are calculated using the pairing fluctuation theory without the particle-hole channel effect. As p increases, the peaks becomes broader and smeared out, and the upper bound in ν beyond which $\chi''_{\text{ph}}(\nu, p)$ vanishes increases towards infinity. In addition, the well defined gap at $p = 0$ in $\chi''_{\text{ph}}(\nu, p)$ near $\nu = 0$ gradually disappears.

towards infinity. At the same time, the peaks in the real part becomes broader and smeared out. From Fig. S5(a), we can see that at $\nu = 0$, the real part slowly increases with p.

The zero frequency value $\chi'_{\text{ph}}(0, p)$ is plotted in Fig. 3 in the main text as a function of p.

III. ANGULAR AVERAGE OF THE PARTICLE-HOLE SUSCEPTIBILITY IN THE BEC REGIME

Finally, we show in Fig. S6 the angular average of the on-shell particle-hole susceptibility, $\langle \chi_{\text{ph}}(0, p = |\mathbf{k} + \mathbf{k}'|) \rangle$ at $\nu = 0$ as a function of momentum k/k_{F}, under the condition $k = k'$, calculated at $1/k_{\text{F}}a = 0.5$. The chemical potential is nearly zero, close to the boundary separating fermionic and bosonic regimes. In comparison with the unitary case shown in Fig. 5 in the main text, we conclude that both dressed and undressed particle-hole susceptibility exhibit stronger temperature and k dependence. Here the small chemical potential determines that the susceptibility

is also much smaller. It is worth mentioning that the level 1 average of the undressed particle-hole susceptibility actually shows a much stronger temperature dependence. This is because $1/k_\mathrm{F}a$ is very close to the fast shut-off shown in Fig. 6 in the main text.

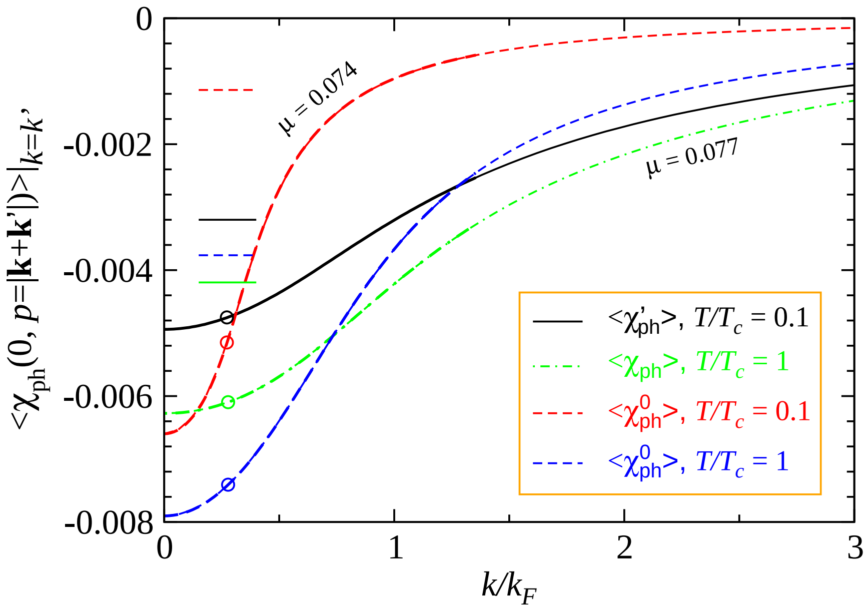

Figure S6. Angular average of the on-shell particle-hole susceptibility, $\langle\chi_\mathrm{ph}(0, p = |\mathbf{k}+\mathbf{k}'|)\rangle$ at $\nu = 0$ as a function of momentum k/k_F, under the condition $k = k'$, calculated at $1/k_\mathrm{F}a = 0.5$. The conventions and legends are the same as in Fig. 5 in the main text.Here $T_\mathrm{c} = 0.226E_\mathrm{F}$ and the chemical potential $\mu/E_\mathrm{F} = 0.077$ and 0.074 at T_c and $0.1T_\mathrm{c}$, respectively. Clearly, there are even stronger temperature and k dependencies in both $\langle\chi_\mathrm{ph}(0, p)\rangle$ and $\langle\chi^0_\mathrm{ph}(0, p)\rangle$ than the unitary case shown in Fig. 5 of the main text.The (absolute) values of Level 2 average are substantially smaller than their level 1 counterpart.
